# What Do New Findings About Social Interaction in Autistic Adults Mean for Neurodevelopmental Research?

**DOI:** 10.1177/1745691620958010

**Published:** 2021-02-09

**Authors:** Rachael Davis, Catherine J. Crompton

**Affiliations:** The Salvesen Mindroom Research Centre, Division of Psychiatry, University of Edinburgh

**Keywords:** autism, development, communication, interaction, neurodiversity

## Abstract

Deficit-based accounts of social and communication abilities continue to dominate autism research. However, emerging findings suggest that this view may be overly simplistic and discount the two-way nature of interaction. Here we discuss the reconceptualization of social cognition to consider such difficulties as examples of bidirectional, multifaceted misattunement between autistic and nonautistic individuals. Aligned with progressive theoretical frameworks, emerging empirical research indicates that mismatches in communication styles can contribute to autistic social difficulties and the important role that nonautistic difficulties play. We highlight two areas of future research with the aim of providing empirical support for the views that the autistic community has proposed over the past 2 decades. We discuss the impact of such a paradigm shift on a number of levels, including how bridging the gap between different interaction styles can reduce stigma and increase understanding. Adopting such a framework will provide radical opportunities for transformative societal changes and education around inclusion.

Autism has traditionally been conceptualized and defined by core deficits in social interaction and communication (American Psychiatric Association, 2013). Research has highlighted that autistic people^1^ perform more poorly than nonautistic people on many measures of social cognition, and these social-cognitive differences are believed to underlie real-world difficulties in interaction (Atherton, Lummis, Day, & Cross, 2019; Morrison et al., 2019).

However, research has also indicated that so-called autism-specific social difficulties could instead be bidirectional in nature and that people of different neurotypes may be mutually misunderstanding one another. An increasing number of studies provide converging evidence of nonautistic people misreading social situations with autistic people. For example, nonautistic people interpret facial emotions less accurately than do autistic individuals (Sheppard, Pillai, Wong, Ropar, & Mitchell, 2016), are less willing to interact with autistic people, overestimate how egocentric autistic people are (Heasman & Gillespie, 2017; Sasson et al., 2017), and overestimate how helpful they are to autistic people (Heasman & Gillespie, 2019b). Nonautistic people are less accurate than autistic people at interpreting the mental states of autistic people (Edey et al., 2016), and finding autistic people difficult to read is related to their being perceived unfavorably by nonautistic people (Alkhaldi, Sheppard, & Mitchell, 2019).

If differences in interaction styles are viewed as impairments for autistic people, we must also consider these differences with nonautistic people as examples of impairments that may exacerbate difficulties in social interactions for autistic people. Whereas the general theoretical narrative of the research literature has focused on autism-specific difficulties in social interaction, two overlapping theoretical frameworks address the mismatch of interaction styles between nonautistic people and autistic people.

First, the double-empathy problem (DEP) proposed by Milton (2012) suggests that miscommunications between autistic people and nonautistic people result primarily from a breakdown in reciprocity and mutual understanding rather than from autism-specific deficits in social communication. Difficulties in interactions occur because of different ways of experiencing the world and processing information (Milton, Heasman, & Sheppard, in press). For instance, autistic and nonautistic people differ in how they process sensory information (Marco, Hinkley, Hill, & Nagarajan, 2011), language (Brock, Norbury, Einav, & Nation, 2008), and social cues (Philip et al., 2010). Researchers of the DEP posit that social communication is not a singular difficulty embodied by a person with autism but a “double problem” experienced by both an autistic person and a nonautistic person within an interaction (Milton et al., in press).

Second, the dialectical misattunement hypothesis (DMH) posits that various psychiatric and developmental conditions are not a disordered function of individual brains but a mismatch of interpersonal dynamics (Bolis, Balsters, Wenderoth, Becchio, & Schilbach, 2017). Drawing on implications from the Bayesian brain hypothesis (Lawson, Rees, & Friston, 2014; Pellicano & Burr, 2012) and predictive-coding and active-inference accounts (e.g., Mirza, Adams, Friston, & Parr, 2019), this framework suggests that autism should be considered a cumulative misattunement between people. Rather than viewing communicative difficulties in isolation, the DMH proposes that an accumulation of repeated reciprocal and dynamic misattunements over time will inevitably lead to increasing divergences in communication styles and interactions between nonautistic people and autistic people.

To test these hypotheses, researchers are exploring the mismatch of communication and interaction styles in a new way: by directly comparing how nonautistic people and autistic people interact with different neurotypes (Crompton, Hallett, Ropar, Flynn, & Fletcher-Watson, 2020; Crompton, Ropar, Evans-Williams, Flynn, & Fletcher-Watson, 2020). Empirical findings support the idea that autistic people interact more successfully and are more comfortable with other autistic people than with nonautistic people, indicating that there may be autism-specific social behaviors underlying more effective autism–autism interactions (Crompton, Hallett, et al., 2020; Crompton, Ropar, et al., 2020; Heasman & Gillespie, 2019a).

This groundbreaking area of research is beginning to provide empirical evidence to support the views that members of the autism community have advocated for many years. Autistic people often highlight feelings of comfort and relaxation, together with unique ways of engaging with each other, when exclusively in the company of other autistic people (Crompton, Hallett, et al., 2020). Many autistic adults have spoken about the transformative impact of finding other autistic people to socialize with: “Being autistic in shared autistic space may be easier than being autistic in nonautistic space or in one’s own personal space” (Sinclair, 2010, para. 30). Autistic adults have reported being better able to predict the behavior of other autistic people and having increased understanding with them (Milton, 2012). Peer support for autistic people and self-advocacy networks provide opportunities for autism-specific social interaction and community building. This can create vital support and understanding for autistic people, who have spoken about the value of sharing experiences and knowledge and the comfort of meeting like-minded people (Crompton, Hallett, et al., 2020).

However, the mechanisms that underlie this ease in autism-specific interaction are not yet known. A key priority for researchers is to better understand these mechanisms and to examine how they may be different from the mechanisms that underlie successful nonautistic interactions and autistic–nonautistic interactions. There are a number of reasons why this is important for research, practice, and the public understanding of autism. Adopting a framework that combines principles of neurodiversity and rigorous scientific methods is essential for reframing social cognition to include the strengths of autistic people and to create new definitions for understanding autism-specific communication and interaction. This will allow us to begin moving beyond deficit-based theoretical accounts of autism that have historically dominated the field of research (Fletcher-Watson & Happé, 2019). Offering empirical support for the idea of difference, not deficit, will contribute to the progression of the rights of autistic people and will have important implications for practice and the public understanding of autism (Cage, Di Monaco, & Newell, 2019).

We propose two priority areas for developing this field of research: building an understanding of the mechanisms behind social development in autism and creating standardized assessments that are sensitive to change and the growth of social skills in autistic people.

## Understanding Developmental Mechanisms

The DMH predicts that gaps in communication styles among nonautistic people and autistic people increase over time, resulting in higher levels of misunderstanding within interactions over development. But what contributes to this widening gap in understanding? To answer this question, research (where possible) must first consider the developmental trajectories of social mechanisms, capturing developmental processes rather than static states, for example, comparing autistic participants to typically developing (TD) groups at a single point in time. Most research on autism and social cognition has traditionally adopted cross-sectional methods; however, findings from longitudinal studies across developmental-disorder research consistently highlight the difficulties of overlaying findings from adults, or one time point, onto developmental processes (e.g., Bishop, 1997; Karmiloff-Smith, 1997, 1998). Although adopting adult frameworks can be valuable when considering end-state development, understanding dynamic changes in behavioral and cognitive profiles across development will be imperative for understanding the age at which these misattunements begin to emerge, how they change over time, and the causal mechanisms underlying communication styles in autism. Furthermore, identifying autism-specific social-communicative developmental trajectories rather than viewing development in autism in terms of divergences from typical development can help us to better understand the factors that contribute to social development and later-life outcomes for autistic people.

In addition to changes in methodological approaches, it is important to acknowledge factors that may specifically underpin neurodiverse social-cognitive development. A complex interplay of cognitive and environmental factors underpin the development of social cognition in TD children (Kilford, Garrett, & Blakemore, 2016). However, there may be differences in which factors play critical roles in social development between children with autism and TD children. For example, cognitive factors that are known to contribute to nonautistic social development include language skills (Fitch, Huber, & Bugnyar, 2010), executive functions (Blakemore & Choudhury, 2006), and IQ (Pellegrini, 1985). Autism-specific environmental factors that could contribute to social development include parental knowledge and understanding of autism (e.g., Green et al., 2010), experience in mainstream or specialist education settings, and experience of peer-support systems for autistic people. It is important to have the scientific infrastructure to chart these developments and to build an understanding of autism-specific social-development trajectories and the mechanisms that underlie them.

Implementing developmental research that includes autism-specific factors could have a real-world impact. For example, researchers are beginning to understand some of the difficulties that many autistic people face regarding social inclusion and the impact of camouflaging (e.g., using explicit techniques to hide behaviors associated with autism and hiding social difficulties) on mental health outcomes (Hull et al., 2017). One important question from a developmental perspective is the extent to which being in an autism-specific environment (i.e., an environment in which autistic people interact with other autistic people) promotes more enriching school or life experiences, reduces camouflaging, and reduces isolation. Research such as this could have a positive impact on areas such as education, health, and social-care settings, which have been identified as priority research areas by autistic people (Cusack & Sterry, 2016).

## Standardized Assessments

To facilitate this type of developmental research, it is crucial to design measures of social cognition that are sensitive specifically to the social and communication skills of autistic people. The social-cognition assessments used in research are currently based on nonautistic social interactions and norms (Morrison et al., 2019). Although emerging research shows that autistic people can interact as efficiently as nonautistic people (although their interaction styles may not conform to nonautistic norms), this directly contradicts findings from social-cognitive-deficit accounts. It is therefore unsurprising that autistic people often perform significantly more poorly than nonautistic people on frequently used social-cognition measures. Furthermore, it is increasingly evident that performance of autistic people on measures of nonautistic social cognition are unlikely to accurately predict a person’s real-world functional and social skills (Sasson, Morrison, Kelsven, & Pinkham, 2020). One key example comes from research focusing on theory of mind (ToM) abilities. ToM tasks are still widely used to measure the ability to attribute mental states to others and the association with other aspects of social-communicative functioning (Gernsbacher & Yergeau, 2019). However, some authors argue that ToM-based tasks do not fully represent the abilities of nonautistic people or autistic people because all tasks are centered around mental states derived from nonautistic people (see Livingston, Carr, & Shah, 2019; Gernsbacher & Yergeau, 2019). We suggest that future assessments should aim to elucidate whether autistic ToM abilities are more successful among other autistic people and whether social difficulties for people are due, in part, to nonautistic difficulties in understanding autism-specific mental states.

It should therefore be a priority for researchers to create a set of autism-specific and autism-inclusive assessments that are coproduced with members of the autistic community from a wide range of backgrounds. This set of assessments would ensure that they assess an accurate conceptualization of successful social interactions from autistic people. These measures could be used to explore the developmental trajectory of the social skills of autistic people and the impact of environment and cognitive skills as described above.

## Toward a New Theoretical Understanding of Autism and Social Interaction

Understanding the underlying differences in communication for autistic people is an essential step in bridging the interaction gaps and understanding between different neurotypes. Conceptualizing communication differences across neurotypes as bidirectional while at the same time acknowledging that autistic people face varying communication difficulties provides a valuable opportunity for future research to influence ways of thinking about differences at a societal level. For example, the limited research that exists on the interactions between different neurotypes suggests that additional social difficulties can be exacerbated by negative perceptions and judgments made by nonautistic individuals (Cage et al., 2019; Sasson et al., 2017). It is also possible that increased familiarity and understanding reduces prejudice and enforces positive effects in terms of interactions. Alleviating the pressure placed on autistic people to move toward “typical cognitive functioning” can reduce stigma, promote inclusivity, and embrace the individual (Bolis, Balsters, Wenderoth, Becchio, & Schilbach, 2017).

Our understanding of autism is changing, with increasing evidence suggesting that social difficulties are at least in part bidirectional. By understanding the mechanisms behind positive autism-specific interactions, we can make a real-world difference on both the support and practice autistic people receive and the public understanding of autism. Bringing neurodiversity to the forefront of research by implementing richer, inclusive methodologies and participatory approaches could provide a bold reconceptualization of social abilities in neurodivergent individuals. Challenging the status quo of social cognition could lead to a paradigm shift in our understanding not only of autism but also a range of neurodivergences and highlight the need to consider how we describe and measure other psychologically defined conditions. Recognizing and embracing the neurodiversity model within scientific research and adopting research frameworks that focus on difference, not deficit, allows the research community to explore meaningful questions that will improve the lives of neurodivergent people (Kapp, Gillespie-Lynch, Sherman, & Hutman, 2013). Crucially, this includes translational work that provides opportunities for effectively supporting autistic people in education, health, and social care.

Charting social development over time, conceptualized within a framework of strengths, could revolutionize the way we understand social interaction in autism and other neurodevelopmental conditions. This research framework creates opportunities for exploring new and exciting hypotheses and novel methodologies and supports and promotes a framework that members of the autism community have advocated for many years.

## Transparency

*Action Editor:* Laura A. King

*Editor:* Laura A. King

## Notes

1. There is debate regarding the language used when describing autism. In this article we use identity-first language (e.g., “autistic person”) as opposed to person-first language (e.g., “person with autism”) to respect the preference of the majority of autistic people (see Gernsbacher, 2017; Kenny et al., 2016).
